# Ungual hyalohyphomycosis caused by *Fusarium cugenangense*

**DOI:** 10.1002/ccr3.2941

**Published:** 2020-11-05

**Authors:** Miki Hirose, Hiromitsu Noguchi, Tadahiko Matsumoto, Utako Kimura, Masataro Hiruma, Rui Kano, Takashi Yaguchi, Norihiro Fujimoto, Takahiro Satoh, Hironobu Ihn

**Affiliations:** ^1^ Department of Dermatology National Defense Medical College Saitama Japan; ^2^ Noguchi Dermatology Clinic Kumamoto Japan; ^3^ Ochanomizu Institute for Medical Mycology and Allergology Tokyo Japan; ^4^ Department of Dermatology Juntendo University Urayasu Hospital Chiba Japan; ^5^ Department of Veterinary Dermatology Nihon University College of Bioresource Sciences Kanagawa Japan; ^6^ Division of Bio‐resources, Medical Mycology Research Center Chiba University Chiba Japan; ^7^ Department of Dermatology and Plastic Surgery, Faculty of Life Sciences Kumamoto University Kumamoto Japan

**Keywords:** efinaconazle, *Fusarium cugenangense*, *Fusarium oxysporum* species complex, nondermatophyte onychomycosis, ungual hyalohyphomycosis

## Abstract

*Fusarium* onychomycosis is uncommon in the temperate climate zone of Japan. Based on the morphological characteristics and a gene analysis, we diagnosed a patient with ungual hyalohyphomycosis caused by *Fusarium cugenangense* belonging to the *F oxysporum* complex. This intractable disease was cured by 6‐month treatment with efinaconazole 10% solution.

## Graphical abstract

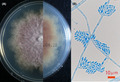

## 1 INTRODUCTION

Members of the genus *Fusarium*, which are ubiquitous soil inhabitants and plant‐pathogenic moulds, cause superficial and invasive opportunistic infections in humans. More than 70 human‐pathogenic *Fusarium* species primarily belong to 8 species complexes. Among these, the *Fusarium solani* and *F oxysporum* species complexes (ie, FSSC and FOSC) account for approximately 60% and 20% of all *Fusarium* infection cases, respectively.^1^ Onychomycosis caused by *Fusarium* species is one of the common nondermatophyte onychomycoses in South America but not in temperate regions.^2^ We herein report a Japanese case of ungual hyalohyphomycosis caused by *F cugenangense,* a member of the FOSC, that was successfully treated with the topical application of an efinaconazole solution.

## 2 CASE REPORT

**Figure 1:**
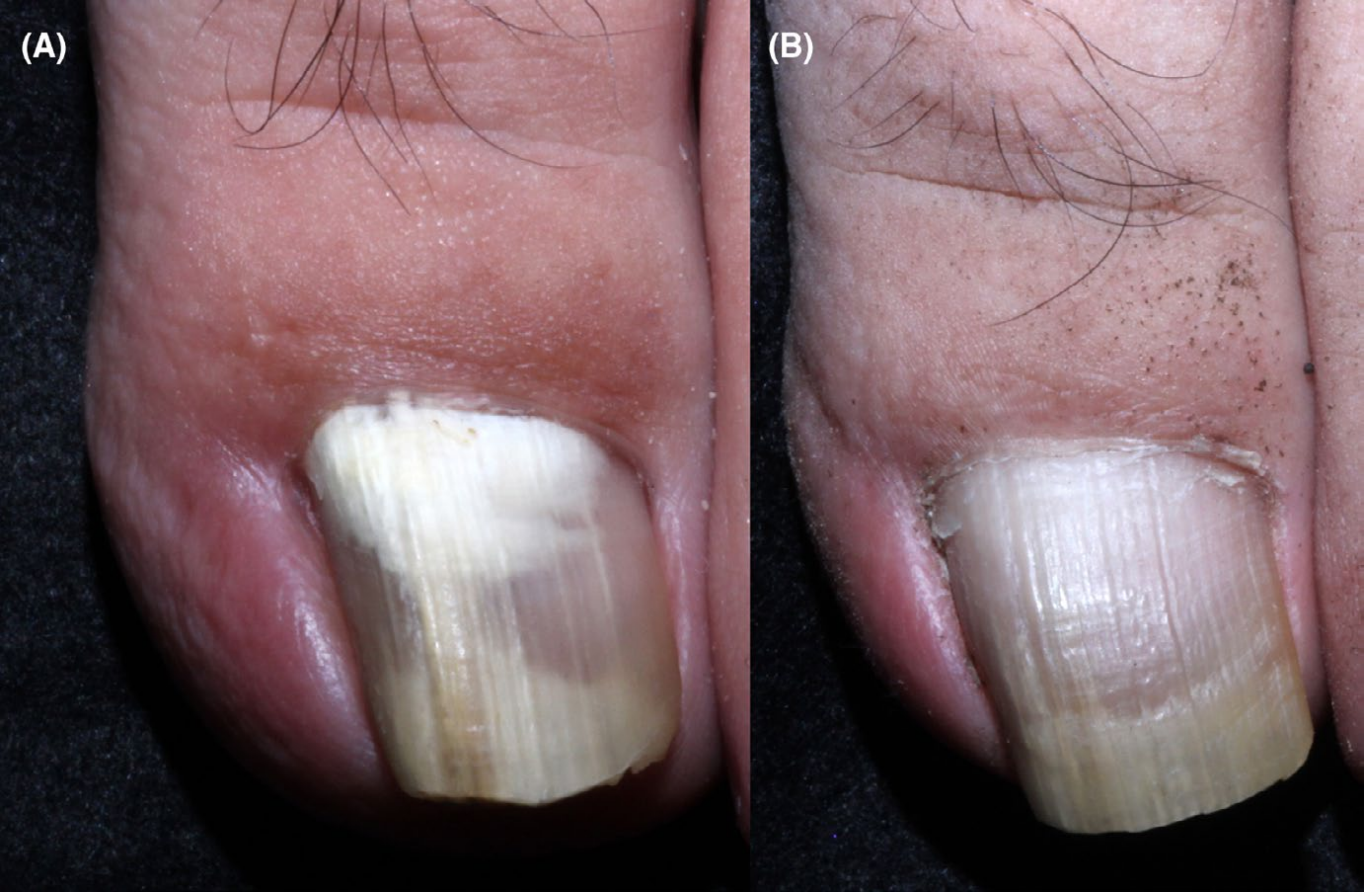
A, Proximal subungual onychomycosis with paronychia on the left big toenail. B, Clinical and mycological cures were attained in 12 mo

A 45‐year‐old male construction worker in Kumamoto, Japan, presented in January 2017 with a whitish discoloration at the base of his left big toenail and painful paronychia that he had first noticed 1 month previously (Figure 1A). His father was diabetic. He had also been treated with ipragliflozin in combination with metformin for type 2 diabetes since 2015. Because he did not comply with his treatment, his blood glucose fluctuated and was uncontrolled (hemoglobin A1c level, 6.8%). His complete blood count was within normal range, and an HIV test was negative. Blood chemistry tests showed slightly increased liver enzyme levels.

**Figure 2:**
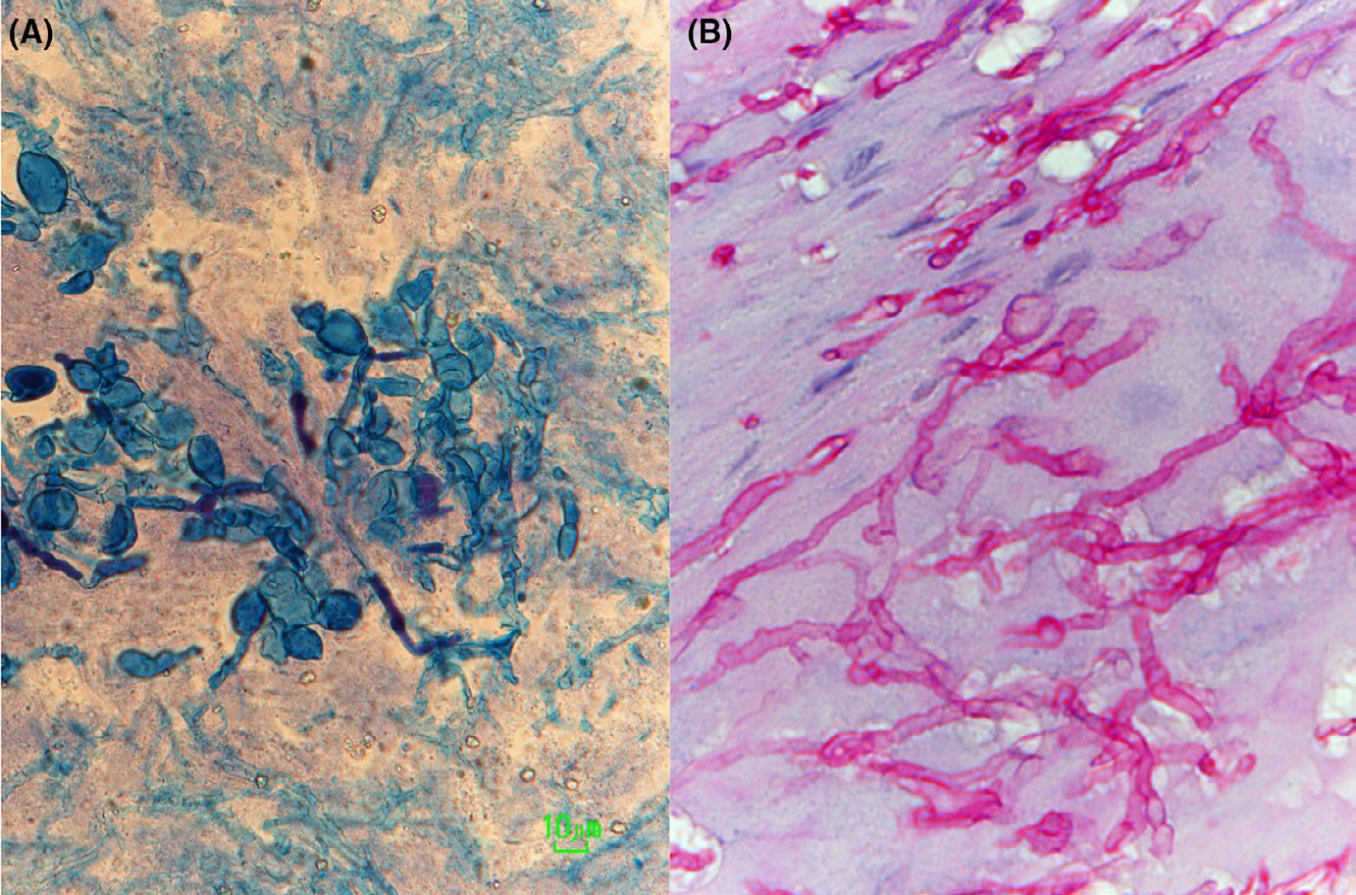
A, Acropetal and intercalary chlamydoconidia (Zoomblue™ fungal staining solution, Hisamitsu Pharmaceutical Co., Tokyo, Japan, original magnification × 400). B, Septate hyphae and chlamydoconidia (periodic acid‐Schiff staining, original magnification × 400)

**Figure 3:**
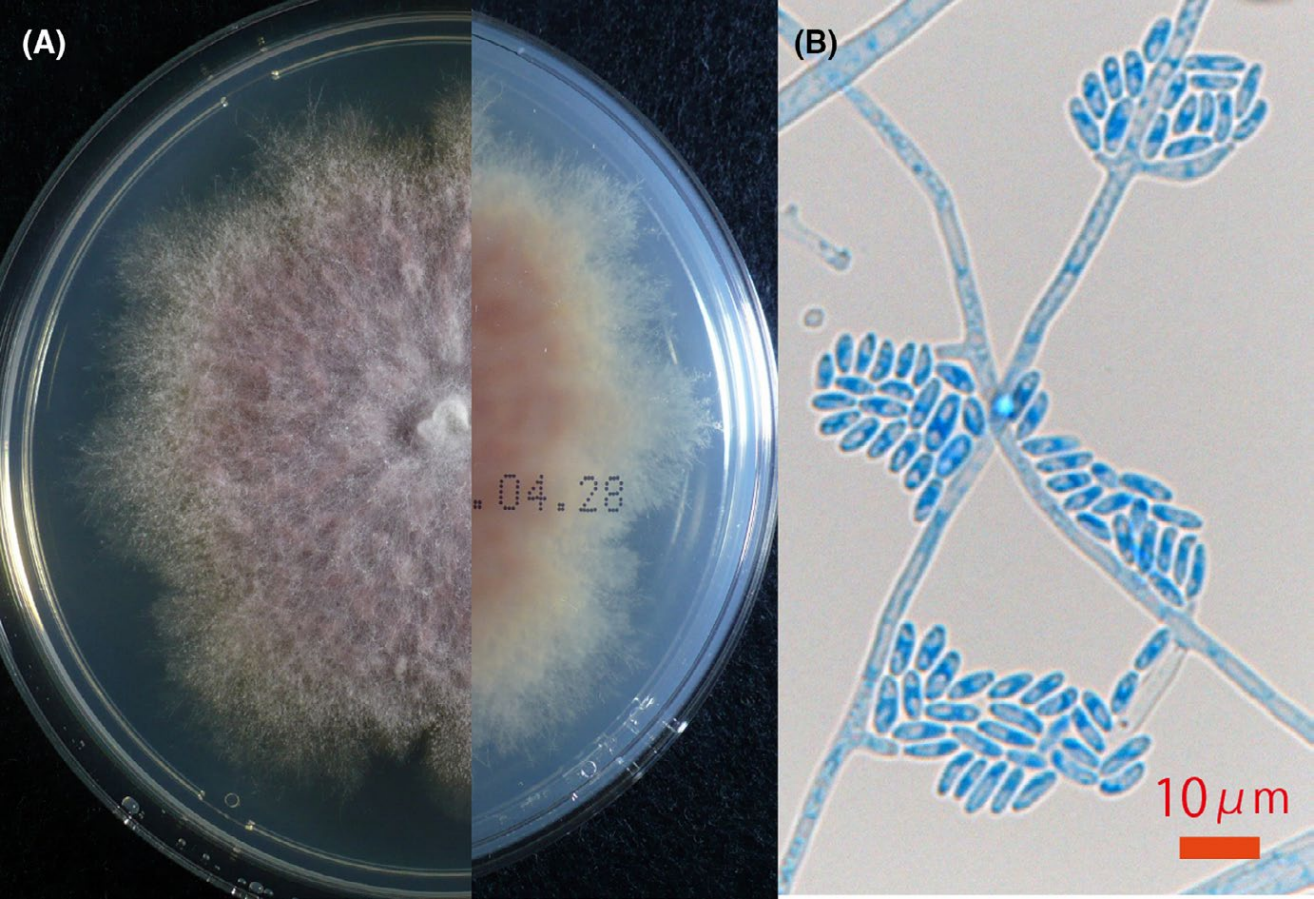
A, Plate culture of *Fusarium cugenangense*, a white floccose colony with pinkish‐gray reverse, on Sabouraud dextrose agar (SDA). B, Slide culture on SDA, showing ellipsoidal microconidia accumulating around the tips of the phialides and along the hyphae (lactophenol cotton blue, original magnification × 400)

A direct microscopic examination of the nail specimen revealed acropetal and intercalary chlamydoconidia (Figure 2A). A histopathological study revealed septate hyphae and chlamydoconidia (Figure 2B). Plate culture on Sabouraud dextrose agar (SDA; Nissui Plate Code 51 033, Nissui Pharmaceutical Co., Ltd., Tokyo, Japan) at 30°C for 3 weeks yielded a whitish floccose to felty colony with a pinkish‐gray reverse (Figure 3A). Slide culture on SDA revealed septate hyphae, conidiophores, phialides, and ellipsoidal microconidia (Figure 3B).

**Figure 4:**
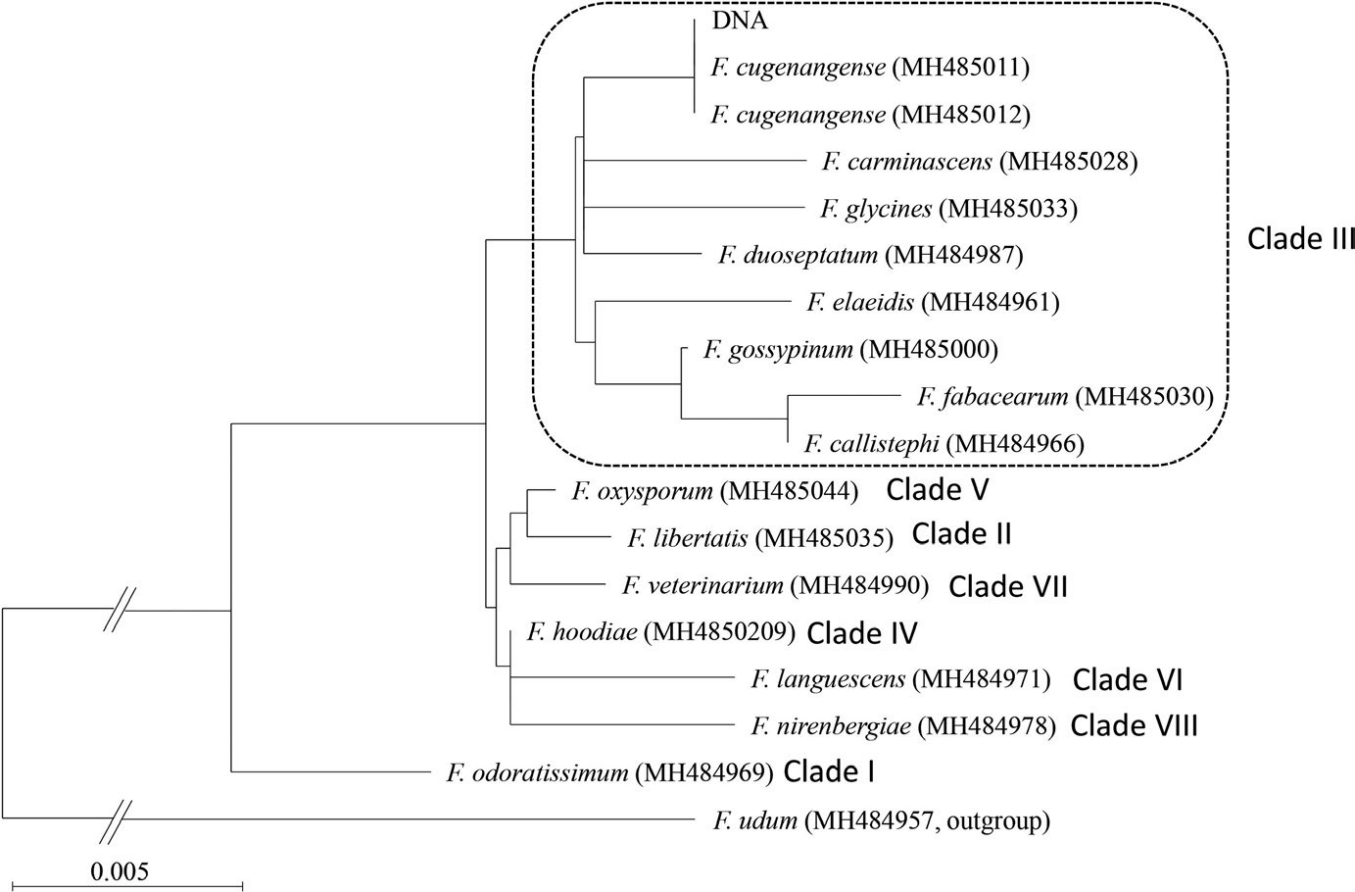
The phylogenic tree of *F oxysporum* species complex on the EF1‐α gene using the neighbor‐joining method

The fungal DNA was extracted using the DNA Extraction Kit, Dr. GenTLE™ (Takara Bio Inc Ltd., Shiga, Japan); the translation elongation factor 1‐alpha (EF1‐α) gene was amplified using the primers EF1 (5′‐ATGGGTAAGGARGACAAGAC‐3′) and EF2 (5′‐GGARGTACCAGTSATCATGTT‐3′)^1^; and the PCR products were purified using the Agarose Gel DNA Purification Kit (Qiagen, Valencia, CA, USA) and sequenced using the BigDye™ Terminator Cycle Sequencing Ready Reaction Kit (Applied Biosystems, Foster City, CA, USA) on an Applied Biosystems 3130 Genetic Analyzer (Applied Biosystems), according to the manufacturer's instructions. The base sequence of the EF1‐α gene had 100% homology (402/402bp) to that of *F cugenangense* CBS 130 304 (GenBank Accession No. MH485012), CBS 130 308 (MH485011), and CBS 131 393 (MH485019). Thus, we identified the isolate as *F cugenangense* N. Maryani, L. Lombard, and Kema et Crous (MycoBank MB826807) (Figure 4).^3^

The patient was diagnosed with ungual hyalohyphomycosis caused by *F cugenangense* using 3 or more out of these 6 major criteria: identification of mould in the nail by direct microscopy, isolation in culture, repeated isolation in culture, inoculum counting, failure to isolate a dermatophyte in culture, and histology.^2^ Antifungal susceptibility testing of the isolate was performed according to the Clinical and Laboratory Standard Institute M38‐A2 protocol. Since the minimum inhibitory concentration (MIC) breakpoints of *Fusarium* species were not established,^4^ we evaluated drug susceptibility referring to the MICs for *Fusarium* species.^5^ The *F cugenangense* strain was susceptible (MICs: ≤2 µg/mL) to amphotericin B, efinaconazole, micafungin, terbinafine, and voriconazole, and was low or less susceptible (MICs: ≥16 µg/mL) to amorolfine, 5‐fluorocytosine, fluconazole, itraconazole, ketoconazole, and miconazole (Table 1). Six‐month treatment with 10% efinaconazole solution cured the disease after 12 months (Figure 1B). As of March 2020, no recurrence of onychomycosis or paronychia has been detected.

**Table 1:** MICs for *Fusarium cugenangense* strain

Antifungals	AMPH‐B	AMF	EFCZ	5FC	FLCZ	ITCZ	KCZ	MFG	MCZ	TRF	VRCZ
MIC (µg/mL)	1	>16	0.5	>64	64	>16	64	0.06	16	2	2
Evaluation	S	L	S	L	L	L	L	S	L	S	S

Abbreviations: 5FC, 5‐fluorocytosine; AMF, amorolfine; AMPH‐B, amphotericin B; EFCZ, efinaconazole; FLCZ, fluconazole; ITCZ, itraconazole; KCZ, ketoconazole; L, low sensitive; MCZ, miconazole; MFG, micafungin; S, sensitive; TRF, terbinafine; VRCZ, voriconazole.

## 3 DISCUSSION

*Fusarium* species are opportunistic pathogens that cause locally invasive cellulitis and disseminated infection in an immunocompromised patient.^6^ However, *Fusarium* onychomycosis usually occurs in healthy individuals in the absence of trauma or dystrophic abnormalities. An ex vivo study showed that *F oxysporum* could invade the healthy human nail, resulting in biofilm formation.^7^ In Japan, 17 cases of ungual hyalohyphomycosis caused by *Fusarium* species (male, n = 9; female, n = 8; mean age, 55.4 years old) have been reported, mainly in immunocompetent individuals (Table 2). Two patients had diabetes, and one had scleroderma. The affected sites were the fingernails, toenails, and both in 4, 11, and 2 cases, respectively. Three patients (17.6%) had paronychia. The pathogens were *F oxysporum*, *F cugenangense*, *F solani*, *F proliferatum*, and *F verticillioides* in five, one, three, three, and three cases, respectively.

**Table 2:** Cases of ungual hyalohyphomycosis caused by *Fusarium* species in Japan

Case No.	Year of report	Age /Sex	Geographic distribution	Underlying disease	Affected site	Subtype	Paronychia	Pathogen	Treatment	Outcome
1	1964	20/F	Shiga	(‐)	R big toenail	N/A	(‐)	*Fusarium oxysporum*	Onychectomy	Cure
2	1969	41/M	Shiga	Tinea pedis	R big toenail	N/A	(‐)	*F oxysporum*	Onychectomy	Almost cure
3	1984	21/F	Kanagawa	Erythermalgia	All toenails	N/A	(‐)	*F oxysporum*	Thermotherapy	Cure
4	1988	81/M	Osaka	Subungual carcinoma	R 1st fingernail	N/A	(‐)	*F oxysporum*	N/A	N/A
5	1997	73/F	Tokyo	Scleroderma	L thumbnail, L 3rd, and R 4th toenails	N/A	(‐)	*Fusarium spp*.	ITCZ	N/A
6	2005	49/M	Tokyo	(‐)	R big toenail	DLSO	(‐)	*Fusarium proliferatum*	ITCZ	Cure
7	2005	54/M	Tokyo	(‐)	L thumbnail and R big toenail	DLSO	(‐)	*F proliferatum*	ITCZ	Cure
8	2005	49/F	Kyoto	(‐)	R and L big toenails	N/A	(‐)	*Fusarium solani*	ITCZ	Improved
9	2006	54/F	Gifu	Poor circulation	R big toenail	DLSO	(‐)	*F oxysporum*	Conservative	Cure
10	2010	58/F	Mie	(‐)	R thumbnail	N/A	(‐)	*F solani*	t‐TBF	Improved
11	2010	57/M	Okinawa	(‐)	R big toenail	DLSO	(‐)	*Fusarium verticillioides*	ITCZ	Failure
12	2011	79/M	Okinawa	(‐)	L big, 2nd, 3rd, and 4th toenails	DLSO	(‐)	*F verticillioides*	t‐TBF	N/A
13	2012	74/M	Kochi	(‐)	R big toenail	PSO	(‐)	*F solani*	ITCZ	Improved
14	2016	73/F	Kumamoto	Diabetes	R big toenail	DLSO	(‐)	*F proliferatum*	t‐EFCZ	Cure
15	2016	50/F	Osaka	(‐)	R thumbnail	PSO	(+)	*Fusarium spp*.	VRCZ	Cure
16	2017	64/M	Ishikawa	(‐)	L 1st fingernail	PSO	(+)	*F verticillioides*	t‐LLCZ	Improved
17	2019	45/M	Kumamoto	Diabetes	L big toenail	PSO	(+)	*F cugenangense*	t‐EFCZ	Cure

Abbreviations: DLSO, distal and lateral subungual onychomycosis; EFCZ, efinaconazole; F, female; F‐RVCZ, fosravuconazole; ITCZ, itraconazole; L, left; LLCZ, luliconazole; M, male; N/A, not applicable; PSO, proximal subungual onychomycosis; R, right; t‐, topical; TBF, terbinafine.

*Fusarium cugenangense* was described in 2019 as a new species causing Fusarium wilt in banana plants. Using multilocus phylogenetic inference and subtle morphological differences with the newly established epitype of *F oxysporum* as a reference point, 15 cryptic taxa were resolved and described as a species, including *F cugenangense*.^3^ Cugenang in Indonesia, where the species epithet is derived from,^8^ has a tropical climate with an average temperature of 19.7°C and annual precipitation of 2,669 mm (1.05 inches) (Köppen climate classification *Af*). Kumamoto is located in the south end of the Japanese archipelago and has a subtropical climate (*Cfa*) with an average temperature of 17.2°C and annual precipitation of 1,986 mm (0.78 inches). Geographically, the southwest Japanese archipelago and the islands of Java are both on the edge of Eurasian plate where many earthquakes and volcanic eruptions occur.

Onychomycosis caused by *Fusarium* species is resistant to terbinafine or itraconazole. Both itraconazole and terbinafine pulse therapies were only partially effective on *Fusarium* onychomycosis, and their clinical cure rates were 52% (13/25 nails) and 50% (4/8 nails), respectively.^9^ Treatment modalities include nail avulsion, surgical debridement, and combination therapy with oral and topical antifungal agents.^10^ An amphotericin B solution (2.0 mg/mL in a 1:1 mixture of DSMO and isopropyl alcohol) was applied in Switzerland.^11^ In Japan, five patients were successfully treated with antifungals (itraconazole, n = 2; efinaconazole, n = 2; voriconazole, n = 1), and the proportion of patients cured with antifungals was 29.4% (5/17 cases).^12^ The MIC of efinaconazole against *Fusarium* species is lower than that of itraconazole.^10^, ^13^ Moreover, efinaconazole shows a broad spectrum of antifungal activities and is expected to be effective for nondermatophyte onychomycosis due to *Candida*, *Aspergillus,* and *Fusarium* species.^13^ The topical efinaconazole is a promising medicine for not only tinea unguium but also nondermatophyte onychomycosis.

## CONFLICT OF INTEREST

None declared.

## AUTHOR CONTRIBUTIONS

M. Hirose, H. Noguchi, and T. Matsumoto: diagnosed the patient, analyzed the data, and wrote the paper. U. Kimura and M. Hiruma: advised on the mycological results. R. Kano: examined the antifungal susceptibility for the isolate. T. Yaguchi: genetically identified the fungus. N. Fujimoto, T. Satoh, and H. Ihn: edited and supervised the manuscript. All authors: discussed the results and commented on the manuscript.
